# Editorial: Privacy-preserving deep heterogeneous view perception for data learning in neurorobotics volume II

**DOI:** 10.3389/fnbot.2023.1151609

**Published:** 2023-02-20

**Authors:** Peng Li

**Affiliations:** School of Software Technology, Dalian University of Technology, Dalian, China

**Keywords:** heterogeneous data, deep learning, privacy-preserving neural computing, deep fuzzy perception, deep multi-view perception

Deep learning has promoted the development of cutting-edge robotic systems with the ability to automatically mine concepts from complex tasks in an open-ended manner. Many novel algorithms and efficient architectures of deep learning with trainable components have achieved remarkable performance in various domains such as machine learning and robotic devices.

Most of the current deep learning methods focus on a single-view perception of objects without fully considering the intrinsic characteristics of data through which objects can be described by heterogeneous views. These heterogeneous views contain complementary knowledge and information that can further improve the representation learning of data. With developments in the easier access of heterogeneous view data promoted by deployments of edge-computing robotic devices, deep heterogeneous-view perceptions distilling knowledge from various views are increasingly attracting more attention. At the same time, heterogeneous view data contains more private information than single view data. Mining large-scale heterogeneous view data inevitably raises the issue of privacy. With the emergence of deep heterogeneous view perceptions, privacies hidden in data are at risk of being leaked more easily. Thus, utilizing the perceptions of deep heterogeneous view data knowledge whilst preserving privacies is also becoming central to neural computing.

This Research Topic collects five high-quality articles reporting the latest applications of privacy-preserving deep heterogeneous learning. Below is a review of the articles published in this collection.

1. The paper by Ma et al. focuses on the privacy protection of robot location-based services. The authors propose a Q-learning particle swarm optimization algorithm in mobile crowdsensing. This new method can make attackers unable to distinguish the specific tasks of users and cut off the association between users and tasks to protect location privacy. The superiority of the method was evaluated on real data.

2. The paper by Lyu and Sun aims to improve the recognition of emotion by robots. The authors introduce a global and local feature fusion method for the recognition of the dance emotion. The new method integrates the global information extracted by long and short-term memory with local information extracted by linear prediction coefficients to enhance the recognition of emotions. This method was evaluated on benchmark data.

3. The paper by Zhang et al. focuses on image classification in robots. The authors propose a novel CapsNet neural network based on the MobileNetV2 structure. The new neural network leverages the dynamic routing mechanism and the attention mechanism for accuracy and robustness. This method was evaluated on the cifar-100 benchmark dataset.

4. The paper by Zhang and Liu aims to improve shadow detection in robots. The authors have designed an unsupervised domain adaptation adversarial learning network with a convolutional block attention module. This new method utilizes hierarchical domain adaption along with boundary and entropy adversarial branches to improve shadow detection. This method was evaluated on the ISTD dataset.

5. The paper by Shen et al. focuses on the recognition of emotion by robots. The authors introduce a linear predictive Meir frequency cepstrum coefficient and bidirectional long short-term memory for recognition of the dance emotion. The new method was evaluated on public datasets and obtained better results compared to six state-of-the-art motion recognition methods.

## Author contributions

The author confirms being the sole contributor of this work and has approved it for publication.

